# Revisiting Key Performance Indicators That Evaluate Food Safety Management Systems: A Short Review

**DOI:** 10.3390/foods14213742

**Published:** 2025-10-31

**Authors:** Ilija Djekic, Nada Smigic

**Affiliations:** Faculty of Agriculture, University of Belgrade, Nemanjina 6, 11080 Belgrade, Serbia; nadasmigic@agrif.bg.ac.rs

**Keywords:** food safety indicators, benchmarks and targets, verification and validation, food processes, food safety culture, food safety 4.0

## Abstract

The main aim of this paper was to revisit scientific manuscripts and identify key food safety performance indicators that may be applicable in different types of food company. A total of 48 papers has been analyzed, and results show that indicators may be categorized as process-based, product-based, and company-based performance indicators. Process-based indicators analyzed the effectiveness of processes found in a majority of food companies such as hygiene, control, maintenance, storage, purchasing, and human resources. Product-based indicators were analyzed through the perspective of production effectiveness as the core process in all food companies. Company-based indicators were deployed in terms of indicators used to verify the effectiveness of the food safety system.

## 1. Introduction

Food companies often intend to certify their food safety management systems (FSMSs) against an international food safety standard, recognized by the Global Food Safety Initiative (GFSI), such as FSSC 22000, BRC and/or IFS [1]. All these standards comprise three parts: prerequisite programs, hazard analysis critical control point (HACCP) system and other food safety-related management and/or company-based requirements. Food systems are considered dynamic and complex, where the use of a ‘system approach’ enables a connection–interaction nexus among its key elements [2]. An essential requirement in every food safety standard, as part of management review, is to analyze the effectiveness of the implemented FSMS, establish its trends, and outline improvement measures [3,4,5]. One of the key methods enabling the evaluation of effectiveness is the development of various performance indicators. They comprise a summary of quantified data providing necessary information on the level of compliance against agreed (food safety) targets [3]. These performance indicators can be related to processes within the company, food products, and the entire food company [4].

Effectiveness in various management systems is analyzed in the context of realizing what is planned and achieving planned results [6]. The time dimension plays a significant role in terms of analyzing FSMSs, where *ex ante* research analyzes the implementation process and *ex post* research explores effects upon implementation and/or certification [7]. To understand the effectiveness of an FSMS, the main approach is to analyze it upon implementation/certification through *ex post* evaluation [8], but with limited investigation of food safety indicators. In parallel, scientific guidance on developing an effective FSMS is still scarce, leaving it to consultants alone to implement FSMS requirements, which is recognized as a barrier due to the (mis)understanding of FSMSs between consultants, food managers, and academia [9,10].

Key Performance Indicators (KPI) can serve as an essential interface between the scientific understanding of food safety and its practical application in the food industry. From a scientific standpoint, KPIs offer quantifiable proxies for assessing FSMS effectiveness, supporting evidence-based decision-making and continual improvement. Conversely, scientific validation provides credibility to KPIs, ensuring that they are not only operationally useful but are also risk-informed and aligned with international standards. This reciprocal value makes FSMS indicators indispensable, both for researchers and industry practitioners aiming to enhance food safety outcomes.

Despite the important role of performance indicators in evaluating FSMSs, scientific guidance on which indicators are most relevant and how to operationalize them remains limited. Therefore, the main objective of this review was to revisit scientific manuscripts and research was to map requirements from major food safety management standards to potential performance indicators, review scientific literature for measurable indicators, and organize them into a structured framework.

## 2. Review Methodology

**Figure 1:**
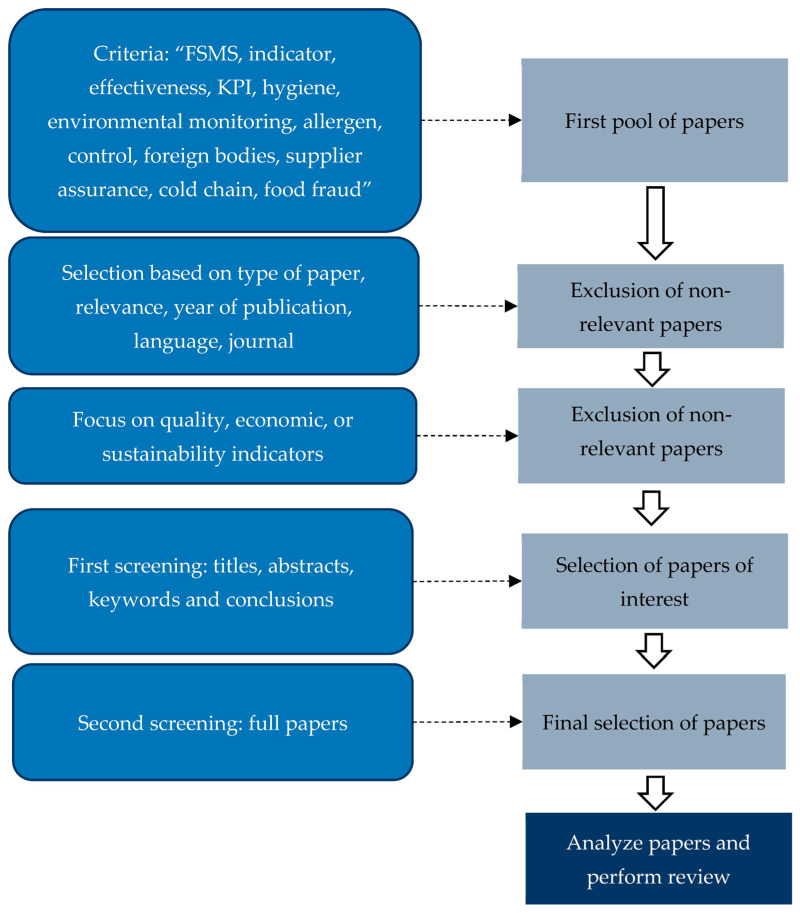
Methodological flow of the review process.

This article was designed with the aim of identifying and structuring performance indicators relevant for evaluating the effectiveness of food safety management systems (FSMSs). The starting point was the analysis of requirements set by major food safety standards, such as ISO 22000:2018, FSSC 22000, BRCGS Food, and IFS Food [3,4,5,11], and regulatory documents, such as Codex Alimentarius and EU hygiene legislation [12,13,14]. These requirements were deployed into potential indicators at the process, product, and company levels. To complement this standards-based framework, a focused review of scientific literature was conducted using combinations of keywords such as FSMSs, indicator, effectiveness, KPI, hygiene, environmental monitoring, allergen, control, foreign bodies, supplier assurance, cold chain, and food fraud. This was performed in Scopus and Web of Science analyzing publications from the period of 2000–2025, focusing specifically on studies proposing or applying measurable food safety indicators. As the number of directly relevant publications was quite limited, a scoping review was performed. The intention of the scoping review was to clarify current concepts and types of research that have been conducted in order to identify literature gaps and identify future research priorities. Only papers that proposed, applied, or critically discussed measurable indicators related to food safety were included. Papers focusing exclusively on quality, economic, or sustainability indicators were not analyzed and as such were excluded. The screening was performed in two phases. In the first phase, screening included the analysis of the titles, abstracts, keywords, and conclusions. In the second phase, the screening included the full reading of the selected papers. Upon completion of the two phases, 48 publications were further included in this analysis. Gray literature, such as industry guidance or company sustainability reports, was used only to illustrate how certain indicators are operationalized in practice. Methodological flow of the review process is depicted in Figure 1.

The three main research perspectives associated with food safety indicators were the food products, processes associated with manufacturing food, and food safety systems in which the companies operate. This concurs with similar research on environmental models in the food chain [15], so the final set of indicators was synthesized into three groups, namely, process-based, product-based, and company-level indicators. Where possible, typical target values and limitations were noted.

## 3. Synthesis of Relevant Literature

### 3.1. Process-Based Performance Indicators

A process-oriented approach and risk-based thinking are seen as necessary tools for the development and implementation of an FSMS, paving the route for improving its effectiveness while producing safe food and complying with applicable legal standards and other food safety requirements [4]. Adopting such an approach includes identifying the processes, understanding their interrelationships, and determining the level of their effectiveness required to achieve intended outcomes [16]. Since food companies differ, depending on their size and complexity, as well as the type of food product(s) they produce, the selection of generic processes related with the prerequisite programs outlined in good hygiene practice [14] was analyzed. These processes include hygiene, control, maintenance, storage, purchasing, and human resources. Production (as the main core process) is explained in terms of product-based performance indicators (Section 3.2). Rationale for this approach is based on two definitions: (i) a process is a “set of interrelated or interacting activities which transforms inputs to outputs”, and (ii) a product is an “output that is a result of a process” [4].

#### 3.1.1. Hygiene

The importance of implementing hygiene procedures together with environmental control measures in food manufacturing is of the utmost significance [17], as these activities act as frontline barriers against the introduction and spread of pathogenic microorganisms, aligned with obeying personal hygiene standards [18]. Their role goes beyond the fulfillment of legal requirements. When hygiene results are tracked and analyzed over time, they can be turned into practical performance indicators. In this way, the hygiene indicators act as early warning signals, pointing to potential weaknesses in the system before they affect the safety of the final product.

One of the most common approaches is monitoring the cleanliness of food contact surfaces (FCSs). Different types of FCS may be found throughout different food industries such as stainless steel, different metals and alloys, glass, wood, paper, ceramic, plastic, silicones, rubber, and elastomers [19], where stainless steel and plastics prevail. The risk of cross-contamination depends on the level of surface contamination and the probability of its transfer to foods [20]. This makes cleaning and disinfection practices an important source of performance data [14]. Besides the type of chemical used, cleaning and sanitation also needs to define “how” cleaning is performed and “where” in terms of different FCSs [9]. It is important to mention that some FSMS standards not only emphasize the need to evaluate the effectiveness of cleaning and disinfection, but also require its validation [21], where BRC standard specifies the identification of target microorganism(s) and hygiene control limits [3].

One of the potential indicators for evaluating process hygiene is obtained by taking swabs from the surfaces and testing them for Aerobic Colony Count (ACC), in accordance with ISO 4833-1:2003 [22]. This is performed either internally in a company laboratory or externally in an accredited laboratory. Based on the obtained results, food companies can classify different cleanliness levels: very clean—Class I (≤1 log10 CFU/cm^2^); moderate cleanliness—Class II (results between 1 log10 CFU/cm^2^ and 2 log10 CFU/cm^2^); and unsatisfactory cleanliness—Class III (results above 2 log10 CFU/cm^2^) [19,23]. These results can be used to define KPIs, for example, “percentage of samples in Class I per month” or “number of unsatisfactory swabs”. In this way, companies can follow trends in their environment and evaluate progress. However, the literature emphasizes that there is no universal cut-off, with proposed thresholds ranging from 0.6 to 1.7 log10 CFU/cm^2^ depending on the type of surface, the food product, or the season [24]. This variability limits the comparison between facilities, but allows companies to follow their own trends and strive for hygiene improvement [25].

The environmental monitoring program (EMP) required by food safety standards [3,11] is another good source of data for measuring hygiene outcomes. Results obtained by taking swabs in different zones of the facility including FCSs, non-food contact surfaces, floors, drains, walls, ceilings, can be used to develop KPIs. Some examples include the “percentage of negative samples”, “distribution of positives across zones” or “time to apply corrective measure following a positive result”. As EMP data often express the detection of pathogens such as *Listeria* spp. or *Salmonella* spp., they are highly relevant and important for early detection of hygienic failures [26,27,28].

The literature shows that EMP is one of the strongest leading indicators of hygiene performance in the food industry [28]. The guidelines for the development of EMPs promote the zone concept, where different zones are defined considering the level of risk contamination or food matrix [29]. Nevertheless, due to differences in sampling strategies and identification of sampling zones, comparisons between industries might be quite challenging, as presented in the review from De Oliveira Mota et al. [28].

Another common practice is the daily/weekly routine inspection of the site (indoor and outdoor), where food companies develop “housekeeping” checklists and visually inspect that the status of internal structures and fittings (floors, walls, ceiling, windows, doors, drainage, personal hygiene facilities and toilets) aligns with good hygiene practice requirements [14]. The development of a quantifiable indicator is usually performed by calculating a “housekeeping score”, where the sum of fulfilled requirements is divided with the total number of requirements listed in the checklist (Equation (1)). While these indicators are more subjective than the results of microbiological methods, they offer the advantages of simplicity, low cost, and frequent application in the food industry.

(1) $$HS\;\left[\%\right]=\frac{Number\;of\;fulfilled\;hygiene\;requirements}{Total\;number\;of\;hygiene\;requirements\;}\times 100\;$$

Allergen management and the prevention of potential cross-contamination is another important requirement addressed in food safety standards [5,11]. Food safety incidents associated with allergen cross-contamination continue to occur [30] and undeclared allergens remain one of the most frequent causes of food recalls [31] with milk, gluten, nuts, eggs, and soy predominating as undeclared allergens. To prevent cross-contamination and minimize risks, food companies mainly define strict hygiene procedures to be applied after the production of products with allergenic ingredients to prevent their occurrence in the next batch. There are two common methods to verify adequate cleaning and sanitation, namely, the testing pH of rinse water to confirm the absence of chemical residues or taking swabs and analyzing the presence of allergen proteins [21]. However, a more advanced approach can be used, known as the voluntary incidental trace allergen labeling (VITAL), to develop indicators [32]. According to this methodology, a food company can calculate the concentration of a protein as a cross-contact allergen using Equation (2):

(2) $$AL\;\left[ppm\right]=RD\;\left[mg\right]\times \frac{1000}{RA\;\left[g\right]}$$

where AL stands for action level, RD stands for reference dose, and RA stands for reference amount. Action level is the calculated concentration of a cross-contact allergen protein to determine whether the allergen should be labeled (as “may contain”) or not. The reference dose is the Eliciting Dose for a specific allergen [33,34], usually referred to as ED05—the dose of allergenic protein that can cause symptoms in 5% of the allergic population [32]. The Reference Amount is typically the serving size of food [32]. The action level is compared with legal requirements that specify target values. Although VITAL was originally created as a decision aid for food labeling, it also shows how the presence of allergens can be quantified and monitored using target values. Translated into metrics-such as “number of batches exceeding VITAL action levels” or “percentage of product lines with validated allergen cleaning procedures”, these measures provide early indications of the adequacy of hygiene programs and highlight their direct impact on consumer protection.

Pest control is also recognized as one of the most important prerequisites for good hygiene practice [14]. Pests mainly cause two types of risks—pathogen transferred from the gut or feces and foreign bodies originating from pests [35]. The most important contaminants from pests are different arthropod fragments, hair and/or feces, which represent over 50% of all physical contaminants found in food [36]. However, due to great differences between food companies (complexity of production, type of raw material and final products, maintenance of building, infrastructure and equipment, location of the company and knowledge of employees), it is a challenge to define a generic indicator [35]. Some food safety standards require monitoring the presence of pests (mainly insects) and evaluate the effectiveness of insect killers, also known as fly-killing devices, by analyzing trends on a monthly basis or in case of an infestation [3]. Companies should, on a case-by-case basis, define their own benchmarks. Possible KPIs include “the average number of pest sightings per month” or “the percentage of traps with positive findings”.

#### 3.1.2. Control

Control, from a food safety perspective, is a process that consists of various measures needed to control microbial, chemical, and physical hazards that are subject to proper validation [37,38]. From a performance perspective, indicators related to control demonstrate whether food safety control measures are functioning as planned. These indicators are closely related to HACCP principles, especially critical control points (CCPs), where monitoring results and deviations trigger corrective actions [14]. In practice, an FSMS relies on a broader set of controls, including operational prerequisite programs (oPRPs) and prerequisite programs (PRPs) as set out in ISO 22000 [4]. Therefore, indicators can be developed at multiple levels, reflecting not only CCP monitoring but also supplier controls, allergen segregation, cleaning and sanitation practices, etc.

One area where KPIs are well established is the control of foreign bodies. Food companies check the presence of metals as foreign bodies, mainly with the help of either metal detectors or magnets [21]. Depending on the approach, a quantifiable indicator can be “less than 1 metal fragment ≥ 2 mm in 100,000 kg of food product”, as outlined in validation standard [37], or “zero” food products with metal fragment when using a metal detector with adequate sensitivity. These indicators are valuable because they provide a clear benchmark for system performance and are relatively easy to monitor. However, the European Rapid Alert System for Food and Feed (RASFF) database shows a variety of non-metal foreign bodies such as bones, glass, pests, plastics, rubbers, stones, wood, etc. [36]. It is not easy to identify them, with one “unique” solution for controlling the presence of all types of foreign bodies. The indicators should be developed on a case-by-case bases, depending on the catalog of potential foreign bodies and measures/devices employed [39]. This illustrates both the strength and the limitation of control KPIs, as they can be precise and measurable, but they are often context dependent. A more technical approach, for example, involves using several magnets on a conveyor, aimed at collecting ferromagnetic particles, and using magnetometers to measure their field strength and capture efficiency. This can serve as an indicator of the effectiveness of the system to control physical hazards.

Trust in the process of control is equally important. Therefore, indicators that assess the reliability of measurement systems in company laboratories play a very important role, since decisions on hazard control depend on the accuracy of laboratory. When a measurement system is not trustful, this can lead to inappropriate decisions and thus to non-compliant (unsafe) products [40]. One of the best-known indicators is the “Z” score value obtained from proficiency testing where a laboratory receives this value as a result of measuring the deviation of its single test results from the true/known value. The “Z” score has three possible outcomes: |z|  ≤  2—results are acceptable and this represents the target value; 2  <  |z|  ≤  3—results are questionable and further analysis is needed; 3  <  |z|—results are unacceptable [41]. In parallel, food companies may perform Gage R&R studies that calculate the variation in the internal measurement system, estimating repeatability (equipment variation) and reproducibility (appraisal variation) [42]. The indicator may be calculated as show in [43] Equation (3):

(3) $$GRR=\;\sqrt{{\left(EV\right)}^{2}+{\left(AV\right)}^{2}}$$

where EV stands for equipment variation and AV for appraisal variation. The target is to achieve GRR ≤ 30% (the measurement system is acceptable when below 10%, while in the range from 10% to 30% it may be acceptable subject to further analysis) [43]. These indicators are critical because they do not directly evaluate the product or process, but the reliability of the data on which the control decisions are based.

#### 3.1.3. Maintenance

The hygienic design of equipment and premises is another essential element of food safety management and is defined as “design and engineering of equipment and premises that are easily cleanable assuring the food is safe and suitable for human consumption” [44]. The design of equipment used in food production has two main roles. It must enable effective cleaning and sanitation, ensuring the microbiological safety and quality of food, and it must prevent negative side effects of chemicals used for cleaning and sanitation and their residues [45]. In terms of achieving this, hygienic design requirements point to the need to use equipment with smooth, nonporous surfaces and rounded edges to reduce the risk of FCSs being hard to clean, resulting in reduced microbial contamination [17]. As a result of rising awareness about this issue, additional food safety requirements outlined in the FSSC 22000 standard require us to perform a risk assessment of the hygienic design of new and/or existing equipment [11].

One of the approaches to developing an indicator for hygienic design may be based on the research of Djekic et al. [45], where a checklist was developed and each requirement was assessed, resulting in full conformity or nonconformity defined as “nonfulfillment of a requirement” [4]. Nonconformity may be ranked as minor or major, depending on the level of nonfulfillment assigned to a weighting factor [46] where the two types of nonconformities are assigned with weights “1” and “2”, respectively, indicating the degree of importance [47,48]. The equation for determining the indicator “level of hygienic design (HD)” considers the number of requirements defined in the checklist (N), the number of minor nonconformities revealed (m), and the number for major nonconformities (M) revealed, as shown in Equation (4):

(4) $$HD=\frac{\left(N-\left((m\times 1\right)+\left(M\times 2\right))\right)}{N}\times 100\left[\%\right]$$

The checklist should include specific requirements outlined in different standards on hygienic design [49,50] and applied across three dimensions [45]: (i) types of materials used for the equipment and their compliance with legislation and relevant standards; (ii) design of equipment and their cleanability potential, i.e., accessibility for inspection, cleaning, and sanitation; (iii) functional requirements focusing on the presence of inconsistent parts such as cavities or niches. Target values should reach 100%.

This indicator has clear strengths. It provides a structured and transparent method for assessing the adequacy of equipment design and allows comparison between equipment/facilities or tracking improvements over time. It also supports continuous improvement by highlighting reoccurring weaknesses in the infrastructure. The use of checklists has the potential to speed up and facilitate the process of error detection, but may lead to subjective judgments [51]. Harmonized tools that are applicable in all food sectors are very rare. In addition, while hygiene design assessments show whether equipment is potentially cleanable, they cannot predict how effectively cleaning will be performed under real operating conditions and therefore need to be cross-checked with hygiene metrics.

#### 3.1.4. Storage

Temperature management is another important indicator for maintaining food safety throughout the food chain [52]. For some food products, an increase in temperature of just a few degrees may lead to significant microbial growth, affecting quality and causing food spoilage with the risk of food poisoning [53]. For this reason, most food companies continuously monitor storage temperatures, often using in-line sensors installed in the storage and transport units. These data are commonly expressed as performance indicators such as the percentage of time that temperatures remain within the target range, the number of deviations recorded per month, or the amplitude of fluctuations around the target temperature.

This target temperature must be set in advance, mainly in line with legislation, although legislation may differ [21]. In some countries, the cold chain is considered to be maintained if food is stored below 5 °C [54,55], while in other it can be up to 7 °C [21] or higher [56]. Moreover, relying solely on average values can be misleading, as short-term temperature fluctuations may go unnoticed, even though they may have an impact on microbial growth dynamics. Therefore, temperature fluctuations need to be validated to detect the occurrence of deviations from prescribed temperatures and to analyze the impact on food safety [21], as temperature fluctuations may stimulate food spoilage and/or the growth of pathogenic bacteria [56].

#### 3.1.5. Purchasing

Purchasing practices are also an important element of FSMS performance as they determine the quality and safety of raw and packaging materials entering the production process. In order to promote the GFSI scheme and the recognition of certain FSMS standards, some requirements stipulate that certified food companies must source their raw materials and primary packaging from suppliers that are certified to some of the recognized FSMS standards [3]. As a result, global food companies are developing measurable indicators outlining the share (or percentage) of suppliers with GFSI-recognized certification and/or share (or percentage) of quantities purchased from this type of supplier [57,58], aiming for a target of 100% (Equations (5) and (6)).

(5) $$GS\;\left[\%\right]=\frac{Suppliers\;holding\;a\;GFSI\;certificate}{Total\;number\;of\;suppliers\;}\times 100\;$$

(6) $$GQ\;\left[\%\right]=\frac{Quantity\;of\;raw\;materials\;from\;suppliers\;with\;a\;GFSI\;certificate\;\left[kg\right]}{Total\;quantity\;of\;raw\;materials\;\left[kg\right]}\times 100$$

GS stands for the share of suppliers holding a GFSI certificate; GQ stands for the share of purchased quantities from suppliers holding a GFSI certificate.

Although these indicators provide clear and quantifiable evidence of control in the supply chain, the certification alone cannot guarantee product safety [59]. In the past, there have been several recalls and incidents at certified companies. This is a further indication that reliance on certification alone can hide emerging risks or weaknesses in suppliers’ practices. For this reason, certification-based KPIs are often supplemented by supplier audits, testing, and risk-based evaluations to provide a complete picture of supply chain reliability [60].

Food fraud is considered to be an economically motivated behavior that compromises food quality and food safety [61]. There are different fraudulent activities, such as intentional dilution, substitution or addition to a food product, as well as unapproved enhancement or mislabeling [3,62]. To combat these practices, food safety standards require a risk and/or vulnerability assessment that mainly targets risks within the supply chain [11]. KPIs that can be identified in this area include the number of fraud incidents detected, the percentage of raw/packaging materials recognized as susceptible to fraud, or the percentage of high-risk suppliers. It is, however, important to note that there are some challenges in this field, such as the hidden nature of fraudulent practices and the variability and reliability of detection methods [63].

#### 3.1.6. Human Resources

When it comes to personnel working in a food company, all standards clearly indicate that they must have adequate competence and awareness of food safety, which implies the fulfillment of various training requirements [3,5]. From a training perspective, there are two main indicators, the number of training hours and the effectiveness of training. As for the first indicator, food companies mainly have their annual training programs and evaluate number of food safety training hours (either total or per employee) with predefined targets for the expected number of training hours. For the second indicator, the best approach is to populate food safety questions and calculate the knowledge score (KS) by dividing the sum of correct answers with the total number of questions, as suggested by Smigic et al. [64] and Smigic et al. [65] (Equation (7)). Target values are set at 100%.

(7) $$KS\;\left[\%\right]=\frac{Number\;of\;correct\;answers}{Total\;number\;of\;questions\;}\times 100\;$$

Some global food companies also share their knowledge across their supply chains by providing specialized food safety trainings and reporting these values in terms of number of hours and/or the number of farmers/suppliers trained [57].

### 3.2. Product-Based Performance Indicators

Unlike process performance indicators, which provide early warnings, product-based indicators only reflect FSMS performance at the end of the chain, when the food has already been produced or is about to reach the market, and so this indicator is linked to production. The most common indicator is the presence of unsafe products, expressed by the number of recalls or withdrawals initiated by a company. To affirm trust, some food companies provide this type of data in their publicly available annual reports [66]. The target values are mainly aligned at reaching “zero” recalls/withdrawal. Another approach is to specify the share (or percentage) of (potentially) unsafe products (UP), as shown in Equation (8):

(8) $$UP\;\left[\%\right]=\;\frac{Quantity\;of\;produced\;unsafe\;products\;\left[kg\right]}{Total\;quantity\;of\;produced\;products\;\left[kg\right]}\times 100$$

In addition to recalls, other product-related KPIs are also used. These include the level of compliance with microbiological criteria (e.g., *Listeria monocytogenes* in ready-to-eat foods), the frequency of exceeding the limit for chemical contaminants (e.g., aflatoxins, pesticide residues), and the rate of consumer complaints linked to food safety issues. Also, the time it takes to complete corrective actions related to recalls might be a useful indicator that provides insight into the responsiveness of the FSMS. Finally, food companies are required to organize recall exercises [11] and the time needed to conduct these types of exercises may be an indicator, as the European Food Safety Authority recommends different timeframes for gathering information, informing relevant stakeholders, public relation activities via social and public media, etc. [67].

Product-based performance indicators are important, as they are objective and closely connected with consumer protection. At the same time, they are reactive, since they highlight problems once unsafe food has been produced or placed on the market. Therefore, these indicators should be interpreted together with process-based performance indicators, such as hygiene monitoring or EMP results, which provide earlier warnings of potential weaknesses in the system. When combined, leading and lagging performance indicators complement each other and provide a complete picture of FSMS performance.

### 3.3. Company-Based Performance Indicators

The commitment to the continuous improvement of an FSMS is mandatory in all standards recognized by GFSI, and as such is also subject to internal and external assessment. Two of the main and most important and common improvement tools outlined in food safety standards are food safety objectives (and evaluation of their fulfillment) and the implementation of corrective actions (and evaluation of their effectiveness) [3,5,11]. Therefore, two indicators can be developed. The first the is “achievement of food safety objectives”, calculated by dividing the number of food safety objectives achieved by the total number of food safety objectives, as shown in Equation (9). The second is “effectiveness of corrective actions” calculated by dividing the number of corrective actions that have been fully implemented by identifying the root cause analysis and preventing recurrence of the identified nonconformities by the total number of corrective actions, Equation (10). In both cases, target values are 100%.

(9) $$FO\;\left[\%\right]=\frac{Number\;of\;achieved\;objectives\;}{Total\;number\;of\;objectives\;}\times 100\;$$

(10) $$EC\;\left[\%\right]=\frac{Number\;of\;effective\;corrective\;actions\;}{Total\;number\;of\;corrective\;actions\;\;}\times 100\;$$

FO stands for the percentage of fulfilled objectives; EC stands for the percentage of effective corrective actions.

### 3.4. Verification of FSMSs

Finally, one of the basic food safety requirements is to verify the effectiveness of the entire FSMS. It usually covers the following: internal audits, the effectiveness of prerequisite programs and HACCP, the analysis of trends related to all testing and sampling (raw materials, final products, water, FCS, personal hygiene), the review of all records that exceeded target values, food safety recall, and incidents and complaints from different stakeholders (consumers, customers, legal authorities) [3,5,11]. One potential indicator can be the use of the FSMS diagnostic instrument [68], which assesses the implementation of over 50 different indicators using a 0–1–2–3 scoring system (“0”—absence of any activity, “1” basic level, “2” average level, and “3” mature level) [69].

However, the main question is whether these benchmarks can be developed and whether the food safety culture provides an “out of the box” perspective [70]. The main reason for incorporating food safety culture is to emphasize the human-organizational building block of food safety, with its main components related to “beliefs and values” or “mission, vision, strategy” [71]. FSMS analysis from a behavioral perspective emphasizes the importance of managing the food safety manners, attitudes, and beliefs of all workers embracing the organizational culture theory [72]. Food safety culture consists of five dimensions, leadership, communication, commitment, resources and risk awareness, and its implementation depends on the country in which the food company operates, as well as the size and type of food produced [70]. To evaluate food safety culture, a useful self-assessment tool is offered to food companies enabling them assessment in four categories: people, process, purpose and proactivity [73]. It provides a scoring system with the target to achieve over 90 points (out of 100).

### 3.5. Critical Analysis and Synthesis

**Figure 2:**
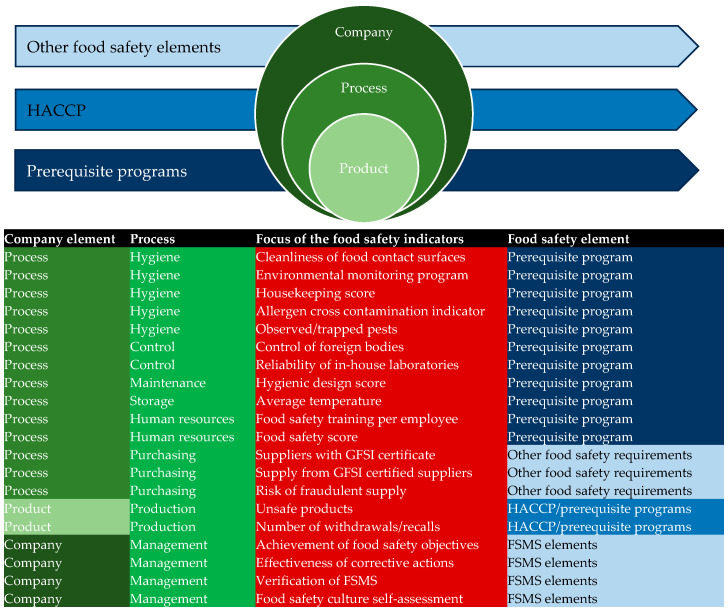
Stacked Venn diagram presenting the deployment of food safety indicators. Legend: GFSI—Global Food Safety Initiative; HACCP—hazard analysis critical control point; FSMS—food safety management system.

The current approach is a combination of (a) a scientific understanding of food safety theory, used to identify risks and develop measurable indicators (mainly related to PRPs and HACCP), and (b) the adaptation of quality-related indicators into food safety indicators (mainly those reflecting compliance with other food safety requirements). It is important to mention that “food safety performance management” is not clearly identified as part of FSMS theory, and this theoretical gap needs to be further studied by food safety scientists. A food safety indicator should be the basis for measuring food safety performance in food companies and authors propose the following definition: “food safety indicator is a measurable representation of the food safety condition or status of processes or products within food safety management systems”. Besides being measurable, all indicators have to be verifiable, repeatable, and technically feasible [74]. As of target values, in most cases it is difficult to define them (like in the case of cleanliness of food contact surfaces) or companies set extreme values located on two sides of the anchor to minimize food safety risks (i.e., “zero” or “100%” as target value) as there are no recommendations set in the literature. This also calls for further guidance. To enable further synthesis and development of food safety indicators, Figure 2 depicts a stacked Venn diagram presenting the deployment of food safety indicators into two dimensions: (i) categorized as product-based, process-based, and company-based; (ii) associated with prerequisite programs, HACCP and other food safety requirements.

Authors believe that, similar to the Food Quality 4.0 approach [75], a triumvirate of three key elements may constitute Food Safety 4.0: (i) food science; (ii) food safety effectiveness; and (iii) Industry 4.0, including different tools such as Internet of Things (IoT), blockchain, and artificial intelligence (AI).

### 3.6. Practical Implications

Results of this study provide some novel insights for both researchers and food safety managers to identify gaps in understanding food safety indicators and assess improvements. They also present one of food safety pillars needed for the quantitative measurement of FSMS effectiveness. As the focus of research spans from supply chains to management, their influence transcends academia and food companies to other stakeholders in the food chain continuum, namely policy makers, food safety inspection services, food safety auditors, customers, and finally consumers.

The food safety indicators explored in this article are not only practical tools for monitoring and improving FSMS performance but are helpful for understanding how food safety systems operate on site and evolve. Also, they enlighten how food safety data-driven insights can support targeted improvement efforts. Their value lies in their ability to make FSMS effectiveness visible, traceable, and comparable. In parallel, the achievement of numerical benchmarks for evaluating food safety performance clearly demonstrates how these measures support evidence-based decision-making in food companies.

## 4. Concluding Remarks and Future Outlooks

When evaluating FSMS indicators, it is important to consider their feasibility and applicability in different contexts. Not all indicators are equally relevant for all food businesses. For example, environmental monitoring for *Listeria monocytogenes* is essential in a business producing ready-to-eat products, whereas it is irrelevant for facilities producing shelf-stable food. Another important factor is the size of the company. Large multinational companies may have the resources to implement advanced monitoring systems, promoting the analysis of samples in accredited laboratories, whereas small and medium-sized companies often have limited financial resources and consequently limited data will be available. In such cases, simpler indicators such as visual hygiene checks or reliance on supplier certifications are often applied. Another challenge is the balance between quantitative and qualitative metrics. Quantitative indicators provide objectivity and comparability, while qualitative or score-based approaches (e.g., hygiene design checklists, food safety surveys) provide valuable insights but are more subjective. These considerations make it clear that FSMS performance indicators should not only be scientifically sound, but also practical, scalable, and tailored to the operational context of the food business.

There are two main limitations of this study. The first is the fact that, due to constraints in findings scientific manuscripts analyzing different food safety indicators, there is a risk that some publications may have been omitted, including manuscripts published in a language other than English and/or gray literature. The second is that quality-related, sustainability-related and/or business-related performance indicators (applicable in food companies) have not been considered as the focus of this research was only to specify food safety-related indicators.

Future efforts should focus on standardizing key indicators, validating their predictive value, and integrating them into wider FSMS maturity and food safety culture frameworks. In parallel, digitalization, real-time monitoring, and use of tools such as IoT, blockchain, and AI will further expand the possibilities for developing food safety performance indicators.

Future studies should focus on analyzing and/or developing food safety indicators related to different food sectors such as food companies producing animal-origin food or plant-based food, food establishments (take away places, restaurants, catering), other food services, etc. This will pave the way for the development of standards/guidelines with specified food safety indicators and target values tailored for different types of food companies. Finally, this set of standards/guidelines will build on current FSMS theory and enable its further development.

## Data Availability

No new data were created or analyzed in this study. Data sharing is not applicable to this article.

## References

[1] GFSI (2022). GFSI Recognized Certification Programme Owners.

[2] Subedi Y.R., Godde C., Korale-Gedara P., Farr J., Fyfe S. (2025). Exploring the Complexity of Food Systems Assessments: A Systematic Literature Review of Frameworks and Indicators. Glob. Food Secur..

[3] BRC (2022). BRC Global Standard for Food Safety.

[4] (2018). Food Safety Management Systems—Requirements for Any Organization in the Food Chain.

[5] IFS (2020). IFS Food, Version 7.

[6] (2015). Quality Management Systems—Fundamentals and Vocabulary.

[7] Djekic I., Tomic N., Pejanovic I., Smigic N., Sredojevic A., Udovicki B. (2025). Food Safety and Environmental Nexus of Fruits Grown under “Zero Residue” Concept. Br. Food J..

[8] Djekic I., Zaric V., Tomic J. (2014). Quality Costs in a Fruit Processing Company: A Case Study of a Serbian Company. Qual. Assur. Saf. Crops Foods.

[9] Djekic I., Tomasevic I., Radovanovic R. (2011). Quality and Food Safety Issues Revealed in Certified Food Companies in Three Western Balkans Countries. Food Control.

[10] Tomasevic I., Smigic N., Djekic I., Zaric V., Tomic N., Rajkovic A. (2013). Serbian Meat Industry: A Survey on Food Safety Management Systems Implementation. Food Control.

[11] (2023). Version 6—Food Safety System Certification, Annex 2: CB Audit Requirements.

[12] European Commission (2004). Regulation (EC) No 852/2004 of the European Parliament and of the Council of 29 April 2004 on the Hygiene of Foodstuffs.

[13] European Commission (2004). Regulation (EC) No 853/2004 of the European Parliament and of the Council of 29 April 2004 Laying Down Specific Hygiene Rules for Food of Animal Origin.

[14] (2022). Rev.5-2022 General Principles of Food Hygiene in Codex Alimentarius Commission—International Food Standards.

[15] Djekic I., Sanjuán N., Clemente G., Jambrak A.R., Djukić-Vuković A., Brodnjak U.V., Pop E., Thomopoulos R., Tonda A. (2018). Review on Environmental Models in the Food Chain—Current Status and Future Perspectives. J. Clean. Prod..

[16] (2015). Quality Management Systems—Requirements.

[17] Chandimali N., Bae J., Hwang J.Y., Bak S.G., Cheong S.H., Lee S.J. (2025). Integrated Hygiene Control Strategies in Food Manufacturing: Technologies, Regulations, and Socioeconomic Impacts. J. Food Prot..

[18] Pakdel M., Olsen A., Bar E.M.S. (2023). A Review of Food Contaminants and Their Pathways Within Food Processing Facilities Using Open Food Processing Equipment. J. Food Prot..

[19] Djekic I., Kuzmanovic J., Andjelkovic A., Saracevic M., Stojanovic M., Tomasevic I. (2016). Microbial Profile of Food Contact Surfaces in Foodservice Establishments. Br. Food J..

[20] Bloomfield S.F., Scott E. (1997). Cross-contamination and Infection in the Domestic Environment and the Role of Chemical Disinfectants. J. Appl. Microbiol..

[21] Djekic I., Smigic N. (2024). How Trustful Are Food Safety Control Measures—Insight into Their Validation. Br. Food J..

[22] (2013). Microbiology of the Food Chain—Horizontal Method for the Enumeration of Microorganisms. Part 1: Colony Count at 30 °C by the Pour Plate Technique.

[23] Djekic I., Kuzrnanovic J., Andelkovic A., Saracevic M., Stojanovic M., Tomasevic I. (2016). Effects of HACCP on Process Hygiene in Different Types of Serbian Food Establishments. Food Control.

[24] De Oliveira A.B.A., Da Cunha D.T., Stedefeldt E., Capalonga R., Tondo E.C., Cardoso M.R.I. (2014). Hygiene and Good Practices in School Meal Services: Organic Matter on Surfaces, Microorganisms and Health Risks. Food Control.

[25] Yoon Y., Kim S.-R., Kang D.-H., Shim W.-B., Seo E., Chung D.-H. (2008). Microbial Assessment in School Foodservices and Recommendations for Food Safety Improvement. J. Food Sci..

[26] Simmons C.K., Wiedmann M. (2018). Identification and Classification of Sampling Sites for Pathogen Environmental Monitoring Programs for Listeria Monocytogenes: Results from an Expert Elicitation. Food Microbiol..

[27] Zoellner C., Ceres K., Ghezzi-Kopel K., Wiedmann M., Ivanek R. (2018). Design Elements of *Listeria* Environmental Monitoring Programs in Food Processing Facilities: A Scoping Review of Research and Guidance Materials. Comp. Rev. Food Sci. Food Safe.

[28] De Oliveira Mota J., Boué G., Prévost H., Maillet A., Jaffres E., Maignien T., Arnich N., Sanaa M., Federighi M. (2021). Environmental Monitoring Program to Support Food Microbiological Safety and Quality in Food Industries: A Scoping Review of the Research and Guidelines. Food Control.

[29] Jackson T. (2014). Management of Microbiological Hazards. Food Safety Management.

[30] Soon J.M., Brazier A.K.M., Wallace C.A. (2020). Determining Common Contributory Factors in Food Safety Incidents—A Review of Global Outbreaks and Recalls 2008–2018. Trends Food Sci. Technol..

[31] Soon J.M., Abdul Wahab I.R. (2021). Global Food Recalls and Alerts Associated with Labelling Errors and Its Contributory Factors. Trends Food Sci. Technol..

[32] VITAL (2024). The Food Industry Guide to the Voluntary Incidental Trace Allergen Labelling Program (VITAL^®^) 4.0. https://vital.allergenbureau.net/wp-content/uploads/2024/08/Food-Industry_Guide_to_VITAL_4.0_2024_F4.pdf.

[33] Mills E.N.C., Adel-Patient K., Bernard H., De Loose M., Gillard N., Huet A.-C., Larré C., Nitride C., Pilolli R., Tranquet O. (2019). Detection and Quantification of Allergens in Foods and Minimum Eliciting Doses in Food-Allergic Individuals (ThRAll). J. AOAC Int..

[34] Houben G.F., Baumert J.L., Blom W.M., Kruizinga A.G., Meima M.Y., Remington B.C., Wheeler M.W., Westerhout J., Taylor S.L. (2020). Full Range of Population Eliciting Dose Values for 14 Priority Allergenic Foods and Recommendations for Use in Risk Characterization. Food Chem. Toxicol..

[35] Djekic I., Kavallieratos N., Athanassiou C., Jankovic D., Nika E., Rajkovic A. (2019). Pest Control in Serbian and Greek Food Establishments—Opinions and Knowledge. Food Control.

[36] Djekic I., Jankovic D., Rajkovic A. (2017). Analysis of Foreign Bodies Present in European Food Using Data from Rapid Alert System for Food and Feed (RASFF). Food Control.

[37] (2008). Guidelines for the Validation of Food Safety Control Measures.

[38] Da Cunha D.T., Hakim M.P., Soon J.M., Stedefeldt E. (2022). Swiss Cheese Model of Food Safety Incidents: Preventing Foodborne Illness through Multiple Layers of Defence. Food Control.

[39] Djekic I., Manning L. (2021). Advances in Techniques for Identifying and Tracking Foreign Bodies in Agri-Food Supply Chains. Burleigh Dodds Series in Agricultural Science.

[40] Plura J., Vykydal D., Tošenovský F., Klaput P. (2022). Graphical Tools for Increasing the Effectiveness of Gage Repeatability and Reproducibility Analysis. Processes.

[41] Medina-Pastor P., Mezcua M., Rodríguez-Torreblanca C., Fernández-Alba A.R. (2010). Laboratory Assessment by Combined z Score Values in Proficiency Tests: Experience Gained through the European Union Proficiency Tests for Pesticide Residues in Fruits and Vegetables. Anal. Bioanal. Chem..

[42] Zanobini A., Sereni B., Catelani M., Ciani L. (2016). Repeatability and Reproducibility Techniques for the Analysis of Measurement Systems. Measurement.

[43] AIAG (2010). AIAG Measurement Systems Analysis (MSA).

[44] EHEDG (2020). EHEDG Glossary.

[45] Djekic I., Tomic N., Smigic N., Udovicki B., Hofland G., Rajkovic A. (2018). Hygienic Design of a Unit for Supercritical Fluid Drying—Case Study. Br. Food J..

[46] Kaydos W. (2020). Operational Performance Measurement: Increasing Total Productivity.

[47] Franceschini F., Galetto M., Maisano D. (2007). Management by Measurement—Designing Key Indicatorsand Performance Measurement Systems.

[48] Djekic I., Dragojlovic S., Miloradovic Z., Miljkovic-Zivanovic S., Savic M., Kekic V. (2016). Improving the Confectionery Industry Supply Chain through Second Party Audits. Br. Food J..

[49] EHEDG (2018). EHEDG Hygienic Design Principles.

[50] (2023). Food Equipment Materials.

[51] Dolberth Dardin F., Stangarlin-Fiori L., Olmedo P.V., Serafim A.L., Opolski Medeiros C. (2019). Elaboration and Validation of a Checklist for the Evaluation of Good Hygiene Practices in Food Trucks. BFJ.

[52] Aung M.M., Chang Y.S. (2014). Temperature Management for the Quality Assurance of a Perishable Food Supply Chain. Food Control.

[53] Carullo A., Corbellini S., Parvis M., Vallan A. (2009). A Wireless Sensor Network for Cold-Chain Monitoring. IEEE Trans. Instrum. Meas..

[54] FSANZ New Zealand (2021). Temperature Control.

[55] FSAI (2018). Temperature Control.

[56] Ndraha N., Hsiao H.-I., Vlajic J., Yang M.-F., Lin H.-T.V. (2018). Time-Temperature Abuse in the Food Cold Chain: Review of Issues, Challenges, and Recommendations. Food Control.

[57] Mondelez (2023). Snacking Made Right. 2023 ESG Report.

[58] Nestle (2023). Creating Shared Value and Sustainability Report 2023.

[59] Powell D.A., Erdozain S., Dodd C., Costa R., Morley K., Chapman B.J. (2013). Audits and Inspections Are Never Enough: A Critique to Enhance Food Safety. Food Control.

[60] Kotsanopoulos K.V., Arvanitoyannis I.S. (2017). The Role of Auditing, Food Safety, and Food Quality Standards in the Food Industry: A Review. Compr. Rev. Food Sci. Food Safe.

[61] Djekic I., Smigic N. (2024). Consumer Perception of Food Fraud in Serbia and Montenegro. Foods.

[62] GFSI (2018). Tackling Food Fraud through Food Safety Management Systems.

[63] Hong E., Lee S.Y., Jeong J.Y., Park J.M., Kim B.H., Kwon K., Chun H.S. (2017). Modern Analytical Methods for the Detection of Food Fraud and Adulteration by Food Category. J. Sci. Food Agric..

[64] Smigic N., Djekic I., Martins M., Rocha A., Sidiropoulou N., Kalogianni E. (2016). The Level of Food Safety Knowledge in Food Establishments in Three European Countries. Food Control.

[65] Smigic N., Antic D., Blagojevic B., Tomasevic I., Djekic I. (2016). The Level of Food Safety Knowledge among Meat Handlers. Br. Food J..

[66] Arla (2023). Annual Report 2023.

[67] EFSA (2023). Best Practice for Crisis Communicators: How to Communicate during Food or Feed Safety Incidents.

[68] Luning P.A., Bango L., Kussaga J., Rovira J., Marcelis W.J. (2008). Comprehensive Analysis and Differentiated Assessment of Food Safety Control Systems: A Diagnostic Instrument. Trends Food Sci. Technol..

[69] Durme J.V., Spagnoli P., Doan Duy L.N., Lan Nhi D.T., Jacxsens L. (2024). Maturity of Food Safety Management Systems in the Vietnamese Seafood Processing Industry. J. Food Prot..

[70] Tomasevic I., Kovacevic D., Jambrak A., Zsolt S., Zotte A., Martinovic A., Prodanov M., Solowiej B., Sirbu A., Subic J. (2020). Comprehensive Insight into the Food Safety Climate in Central and Eastern Europe. Food Control.

[71] Spagnoli P., Vlerick P., Jacxsens L. (2023). Food Safety Culture Maturity and Its Relation to Company and Employee Characteristics. Heliyon.

[72] Tomasevic I., Kovacevic D., Jambrak A., Szendro K., Zotte A., Prodanov M., Solowiej B., Sirbu A., Subic J., Roljevic S. (2020). Validation of Novel Food Safety Climate Components and Assessment of Their Indicators in Central and Eastern European Food Industry. Food Control.

[73] BRC (2022). BRC Culture Excellence—Food Safety Culture Module.

[74] Djekic I., Tomasevic I., Muthu S.S. (2019). Environmental Indicators in the Meat Chain. Quantification of Sustainability Indicators in the Food Sector.

[75] Djekic I., Velebit B., Pavlic B., Putnik P., Merkulov D., Markovinovic A., Kovacevic D. (2023). Food Quality 4.0: Sustainable Food Manufacturing for the Twenty-First Century. Food Eng. Rev..

